# Sexual Dimorphism in Interstitial Lung Disease

**DOI:** 10.3390/biomedicines10123030

**Published:** 2022-11-24

**Authors:** Mari Ozaki, Arlene Glasgow, Irene K. Oglesby, Wan Lin Ng, Sile Kelly, Catherine M. Greene, Laura Durcan, Killian Hurley

**Affiliations:** 1 Department of Medicine, Royal College of Surgeons in Ireland, Education and Research Centre, Beaumont Hospital, D09 YD60 Dublin 9, Ireland; 2 Tissue Engineering Research Group, Royal College of Surgeons in Ireland, D02 YN77 Dublin 2, Ireland; 3 Department of Clinical Microbiology, Royal College of Surgeons in Ireland, D09 YD60 Dublin 9, Ireland; 4 Department of Rheumatology, Beaumont Hospital, D09V2N0 Dublin 9, Ireland

**Keywords:** interstitial lung diseases, connective tissue diseases, sex hormones, X-chromosome, idiopathic pulmonary fibrosis, sexual dimorphism, gender differences

## Abstract

Interstitial lung diseases (ILD) are a group of heterogeneous progressive pulmonary disorders, characterised by tissue remodelling and/or fibrotic scarring of the lung parenchyma. ILD patients experience lung function decline with progressive symptoms, poor response to treatment, reduced quality of life and high mortality. ILD can be idiopathic or associated with systemic or connective tissue diseases (CTD) but idiopathic pulmonary fibrosis (IPF) is the most common form. While IPF has a male predominance, women are affected more greatly by CTD and therefore associated ILDs. The mechanisms behind biological sex differences in these progressive lung diseases remain unclear. However, differences in environmental exposures, variable expression of X-chromosome related inflammatory genes and sex hormones play a role. Here, we will outline sex-related differences in the incidence, progression and mechanisms of action of these diseases and discuss existing and novel cellular and pre-clinical studies. Furthermore, we will highlight how sex-differences are not adequately considered in pre-clinical disease models, how gender bias exists in clinical diagnosis and how women are underrepresented in clinical trials. Future action on these observations will hopefully shed light on the role of biological sex in disease development, identify potential targets for intervention and increase female participant numbers in clinical trials.

## 1. Introduction

Biological sex differences play an important role in the development and progression of disease but also in the response to drugs and treatment. Sex differences have been studied at length in diseases such as cardiovascular disease, musculoskeletal disease and neuronal disease [1]. The role of sex in lung diseases, and in particular, in interstitial lung diseases (ILD), is an expanding research area. Although, the complete mechanisms behind these sex differences are unknown, differences between men and women are known to be influenced by variances in male and female biology, epigenetic differences and sex hormones. Understanding these differences are important considerations for patients, pulmonary researchers and clinicians.

Lung development and maturation differ based on biological sex and the influence of sex hormones. Female foetal lungs mature faster than their male counterparts, evidenced by earlier production of surfactant [2]. Male neonates are therefore more susceptible to the development of respiratory distress syndrome (RDS) as it is largely due to surfactant deficiency [3]. The female lung is generally smaller than the male lung at birth with physical differences persisting through to adulthood. Dysanaptic or disproportionate growth of the lung parenchyma and the airways during maturation is observed in both sexes, however, faster large airway versus parenchyma growth is observed in young girls with the opposite effect present in young boys [4]. However, in general, adult males possess larger conducting airways regardless of lung or body size [5].

Numerous studies have identified the role that estrogens and androgens play in lung development, with both stimulatory and inhibitory effects being reported, respectively [4]. Critical roles for estrogen and its receptors, estrogen receptor alpha (ERα) and estrogen receptor beta (ERβ), have been described for alveolar development. Conversely, a negative role for testosterone on surfactant, (a complex made up of phospholipids and protein that prevents alveolar collapse) production has been reported in several species [5].

In this review, we aim to summarize the existing literature on sex-specific differences in the development and progression of ILD, including idiopathic pulmonary fibrosis (IPF), connective tissue disorders (CTD) associated with ILD, and other pulmonary fibrotic diseases with a focus on autoimmunity. We discuss the current understanding of the role of sex in cellular and pre-clinical studies in the hope of better identifying the potential mechanisms responsible. We further examine gender differences and bias in human trial participants in clinical studies in ILD. Improved understanding of the critical role of sex differences in disease development is fundamentally important for pulmonary researchers and clinicians alike, helping to identify potential therapeutic targets and direct future research in this much needed field of investigation.

## 2. Interstitial Lung Diseases

ILDs are a group of heterogeneous progressive pulmonary disorders that are characterised by tissue remodelling and/or fibrotic scarring of the lung parenchyma. ILD patients often experience lung function decline with advancing symptoms, poor response to treatment and reduced quality of life. ILDs can occur due to genetic, physical or environmental factors, but some are idiopathic. Many are associated with various systemic diseases, and are classified according to specific clinical, radiological and histopathological features. The most common form of ILD is IPF but there are a large number of ILDs that are associated with CTDs.

## 3. Pathogenesis of Pulmonary Fibrosis

Pulmonary fibrosis is a dysregulated wound healing process in which injured epithelium is not adequately repaired. It is characterised by fibroblast accumulation and the excessive deposition of extracellular matrix (ECM), resulting in the destruction and remodelling of the normal lung architecture, leading to the loss of lung function and finally death. Although the aetiology of pulmonary fibrosis is still poorly understood, numerous risk factors and predisposing factors have been proposed. Pulmonary fibrosis is generally accepted to be triggered by repeated subclinical injury to endothelial and alveolar epithelial cells, caused by an interaction between genetic predisposition, aging and environmental agents such as cigarette smoking (CS), gastroesophageal reflux (GER), viruses and exposures to metal, wood and silica dusts, reviewed extensively in [6,7,8].

**Figure 1:**
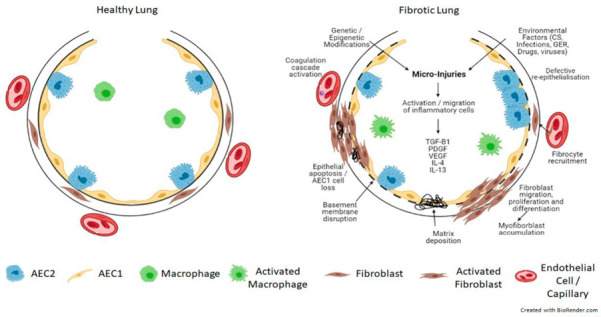
Schematic view of the pathogenesis of pulmonary fibrosis. Genetic and epigenetic factors in combination with repeated injury from environmental factors to the lung alveolar epithelium result in epithelial and endothelial cell damage and the disruption of the basement membrane. This initiates an inflammatory response and the secretion of pro-fibrotic cytokines such as TGF-β, PDGF, VEGF, IL-4 and IL-13. This results in the activation of the coagulation cascade, abnormal vascular remodelling and repair and aberrant epithelial-mesenchymal crosstalk, together with the recruitment, migration and differentiation of fibroblasts/fibrocytes into myofibroblasts. These myofibroblasts are resistant to apoptosis and accumulate, resulting in extracellular matrix deposition and the formation of fibrotic lesions, which leads to progressive lung remodelling and architectural distortion. TGF-β = transforming growth factor beta; PDGF = platelet-derived growth factor; VEGF = vascular endothelial growth factor; IL-4 = interleukin 4; IL-13 = interleukin 13. Created with BioRender.com.

Endothelial injury results in the activation of platelets, coagulation pathways, fibrin-rich clot formation, vasculogenesis and angiogenesis. Alveolar epithelial cell injury and apoptosis progressively disrupt the lung matrix, interfering with the integrity of the basement membrane, resulting in the activation and accumulation of myofibroblasts. Chemokine gradients recruit inflammatory cells, resulting in the infiltration of neutrophils, eosinophils, lymphocytes, and macrophages. The inflammatory cells then secrete chemokines, cytokines and growth factors, such as IL-4, IL-13, and TGF-β. These cytokines display significant pro-fibrotic activity, and act by recruiting, activating and proliferating fibroblasts, macrophages and myofibroblasts, collagen synthesis/deposition. In addition to fibroblasts, fibrocytes, a group of circulating cells can also differentiate into myofibroblasts. These myofibroblasts are resistant to apoptotic signals and in combination with the impaired capacity for alveolar epithelial type 2 cells (AEC2) to renew, lead to excess ECM deposition and aberrant alveolar epithelium regeneration and re-epithelialization, preventing injury resolution, resulting in progressive pulmonary fibrosis (Figure 1) (Reviewed in [6,8,9]).

In addition to these mechanisms, others such as reactivation of embryologic pathways, oxidative stress, endoplasmic reticulum stress and the unfolded protein response as well as sex hormones have been proposed [10,11,12] in pulmonary fibrosis pathogenesis.

## 4. Sex Differences in IPF

Mortality rates for IPF are high and increasing according to data from both the United States (US) National Vital Statistics System and the Office of National Statistics in the United Kingdom (UK). Age-adjusted mortality rates for IPF have increased nearly 10% from 18.81/100,000 in 2000 to 20.66 in 2017 in the US and 1.66/100,000 in 1979 to 8.29 in 2016 in the UK with mortality rates higher in men and with increasing age in both cohorts [13,14].

Han et al. 2008 made some important observations namely that males with IPF have a more rapid deterioration of exertional desaturation over time than females; that survival is worse in males compared to females; and that females have a better survival rate after additional adjustment for relative change in exertional desaturation and percentage forced vital capacity (FVC) on spirometry [15]. These findings were in contrast to a lesser-powered retrospective analysis of IPF patients, where sex did not appear to be a significant predictor of adjusted survival [16]. A summary of the epidemiological and clinical sex differences in IPF is shown in Table 1.

IPF is among many fibrotic disorders in which researchers have found effects of sexual dimorphism in terms of prevalence and progression. Women are less likely to develop IPF even in the setting of genetic disease with the same mutations [55]. They also have a better survival rate when they do develop the disease [55]. Similar findings have also been found in other fibrotic associated disorders such as kidney and liver disease. Men with chronic kidney disease have been shown to progress more rapidly than women to end-stage kidney failure requiring dialysis [56] and end-stage liver disease or cirrhosis is more common in men than women [57].

Our understanding of the molecular basis for the male predominance of IPF is currently lacking. However, it is thought that in contrast to the adverse effects of estrogens in chronic inflammatory lung diseases such as asthma and cystic fibrosis, estrogens may be protective against airway fibrosis [58], whilst androgens are detrimental [59]. Curiously, results from experimental fibrosis studies in vivo are species dependent. For example, male mice develop more severe bleomycin-induced pulmonary fibrosis than age-matched females [60]. In contrast, bleomycin instillation to rats is more severe for females versus males, alleviated by ovariectomy, and exacerbated by estradiol replacement [61].

A change in the ratio of estrogen receptor alpha (ERα):ERβ can alter cell function. Interestingly, Elliot et al., 2019 found that ERα expression was upregulated in male IPF lung tissue and fibroblasts at both the mRNA and protein levels, that IPF fibroblasts responded better to estrogen in comparison with controls, and that blocking ER lessened this effect [62]. They also showed in a mouse model of bleomycin-induced fibrosis that pharmacologic inhibition and mimicry of ERα and ERβ, respectively attenuate fibrosis. However, mice with a mutation in the AF2 estrogen ligand-binding domain of ERα still developed bleomycin-induced fibrosis, indicating that other ligands could be responsible for activation of ERα to mediate fibrosis. Furthermore, the authors showed that Insulin-like Growth Factor-1 (IGF-1) was increased in male IPF lung tissue and myofibroblasts, and in lungs from bleomycin treated mice. In myofibroblasts from IPF lungs, IGF-1 stimulated transcriptional activation of a transfected estrogen-responsive element more potently than estrogen stimulation. These data may explain how the ERα receptor mediates fibrosis in a male predominant disease where circulating estrogen levels would be low.

In addition to the effects of sex hormones and their receptors, the loss of the transcriptional repressor ELK1 has been shown to enhance α5β6 integrin (ITGB6) gene expression and fibrosis; this integrin is dramatically increased in IPF. Interestingly, ELK1 is an X chromosome gene and therefore this may potentially contribute to the sex imbalance in IPF [63].

## 5. Connective Tissue Diseases

CTD are a broad group of heterogeneous systemic autoimmune disorders that are characterised by immune-mediated chronic inflammation, often resulting in tissue damage, collagen deposition and abnormal repair, leading to dysfunction of the target organ over time. The majority of CTD can target the lung and are associated with ILD. For example, the estimated prevalence of ILD in patients with systemic sclerosis (SSc), Primary Sjögren’s syndrome (pSS), rheumatoid arthritis (RA) and systemic lupus erythematosus (SLE) are 40%, 30–40%, 40% and 10–12%, respectively [64]. CTD are associated with substantial morbidity and mortality. Although ILD usually develops in patients with known CTD, it can be the first and only indicator of a previously undiagnosed CTD.

CTD affects more women than men and are one of the leading causes of disability in women. A strong female bias is found in the incidence of the CTDs that are most frequently associated with ILD: SSc, SS, SLE and RA [65]. However, despite the increased prevalence of RA and SSc in women, male sex is a risk factor for ILD development in both these CTDs [66,67]. The epidemiological and clinical sex differences in rheumatoid arthritis-associated interstitial lung disease (RA-ILD), systemic sclerosis-associated ILD (SSc-ILD), systemic lupus erythematosus-associated interstitial lung disease (SLE-ILD) and Sjögren’s syndrome-associated interstitial lung disease (SS-ILD) are summarised in Table 1.

Rheumatoid arthritis is a chronic systemic inflammatory disease with incidence and prevalence rates twice as common in women compared with men (Table 1) [18]. In addition to men having a higher likelihood of positive serology and experiencing a later disease onset, men are also more likely to develop features such as ILD and subcutaneous nodules [68,69]. Patients with RA are estimated to have approximately 10% lifetime risk of a diagnosis of ILD and are almost nine times more likely to be diagnosed with ILD compared to patients without RA [29]. A large multicentre UK study showed an almost equal prevalence of RA-ILD between both sexes despite RA having female predominance [38]. The risk factors for developing RA-ILD are smoking, elevations of rheumatoid factor and anti-citrullinated protein antibodies, presence of rheumatoid nodules, elevated erythrocyte sedimentation rate, longer RA duration and presence of the MUC5B promoter variant [29,70,71,72]. A study by Lee et al. showed that usual interstitial pneumonia (UIP) pattern is more prevalent in male patients with RA-ILD (M:F, 8:2) while non-specific interstitial pneumonia (NSIP) was female dominated (M:F, 0:6) [25].

SSc is an autoimmune CTD with an approximate female-to-male incidence ratio of 3:1 [19,73]. The two leading causes of death for patients with SSc are ILD and pulmonary arterial hypertension (PAH) which affects 80% and 15% of the SSc cohort, respectively [18,74]. Among demographic factors such as African American race and diffuse skin disease, male sex is associated with increased risk of developing SSc-ILD [23,75]. In a study by Peoples et al., disease characteristics associated with female sex included limited cutaneous systemic sclerosis (lcSSc), younger age of onset, anti-centromere antibody with most frequent cause of death being PAH [45]. On the other hand, males displayed older disease onset, were more frequently cigarette smokers, had diffuse cutaneous systemic sclerosis (dcSSc), presence of anti-SCL70 and anti-U3RNP antibodies and ILD was the most common cause of death [45]. Interestingly, anti-SCL70 and anti-U3RNP antibodies have been frequently linked with ILD [43]. Therefore, in clinical practice patients with SSc-ILD should be monitored closely as it may be necessary to initiate immunosuppressive or antifibrotic therapy given the likelihood of disease progression.

People with SLE, a condition associated with a dramatic gender disparity in adults (9:1, female:male), have many reported pulmonary complications including pleural disease, ILD, vasculitis, pulmonary embolism, pulmonary hypertension, large airway disease, shrinking lung syndrome, and infection. Historically, the prevalence of SLE-associated ILD has been suggested to be very low, between 3 and 9% [76,77] although these figures predate high resolution CT. More recent estimates show the prevalence closer to 10% [78] with maintenance of the female predominance seen in adult SLE. Major risk factors for developing SLE-associated ILD include long disease duration, and in particular older age at onset. Overlapping clinical and serological abnormalities with SSc such as Raynaud’s, sclerodactyly and antibodies to SCL70 and RNP [47,48,78,79,80] in addition to the antibodies which overlap with Sjögren’s, anti-Ro (SSA) and anti-La (SSB) are also risk factors. The optimal therapy for SLE-ILD is not known due to a paucity of literature reporting outcomes and the absence of controlled trials. Distinguishing established irreversible damage from acute changes is important, as this directs the need for immunosuppression, whilst the presence of progressive fibrosis makes it more likely that an antifibrotic therapy should be initiated.

Primary Sjögren’s syndrome (pSS) is a systemic autoimmune disease with a female-to-male predominance of 9:1 [21] characterised by lymphocytic infiltration of exocrine glands. Pulmonary involvement is a relatively common extra-glandular phenomenon, affecting 9–22% [35,36,37], with an increasing prevalence with time and a persistent female predominance [81]. Onset of pulmonary symptoms can occur at any time in the disease course; in some it can pre-date other manifestations whilst in others it is a late phenomenon [82]. ILD, which is one of many pulmonary manifestations associated with pSS is more common in smokers, older age, those with hypergammaglobulinemia, elevated rheumatoid factor (RF) or antinuclear antibody titres, positive anti-SSA or -SSB antibodies, increased C-reactive protein (CRP), and reduced serum C3 levels [83,84,85]. Similar to other autoimmune related ILDs, there is a paucity of literature reporting outcomes and an absence of controlled trials in pSS related ILD. Patients with pSS are rarely treated with immunosuppression for other manifestations and so in those with pulmonary involvement, it is usually the severity of lung involvement which directs therapy. Similar to the approach in SLE, distinguishing established irreversible damage from acute changes is crucial, as this directs the need for immunosuppressive versus antifibrotic therapy.

Understanding the molecular mechanisms contributing to the female skewed prevalence of these CTD/autoimmune diseases that commonly exhibit pulmonary manifestations may offer insight into disease progression. Therefore, we now discuss the basis of biological sex differences in SLE, pSS, RA and SSc.

## 6. Sex Hormones and the X Chromosome in Autoimmunity

Females typically have a stronger immune response than males, a phenomenon often regarded as a double-edged sword [86,87]. On one hand, females have an advantage in responding to and resolving acute infections; on the other hand they are more likely to develop an autoimmune disease (AD) including CTDs [88,89]. Sex hormones are one of the obvious sources of endogenous biological differences between males and females. In general, estrogen is immunostimulatory; progesterone and androgens are immunosuppressive. However, the role of estrogen is complex and it can display opposing effects that are dose-dependent. Examples of altered hormone levels and potential mechanisms of action in CTD are shown in Table 2.

Puberty, pregnancy and menopause are three major endocrinological transitions in a woman’s lifespan. These changes can affect both the innate and adaptive immune systems and can influence the development of AD, as reviewed by Desai and Brinton [102]. For example, pregnancy is associated with a shift away from Th1 towards Th2 immune responses to avoid rejection of the fetus and enhance antibody production for maternal transfer [87]. This appears to worsen the antibody-mediated disease SLE, but often improves symptoms in other AD such as RA [88]. Pregnancy has also been suggested to contribute to female AD development due to fetal microchimerism—the transfer of hematopoietic stem cells from fetal to maternal circulation. These cells can establish lineages in the mother and persist for decades, where they could become targeted as foreign cells [103]. However, evidence for the involvement of fetal microchimerism in AD development is conflicting. Whilst some studies found that women with SSc who had delivered at least one son had more male DNA in their blood than controls [104], and Y-chromosome containing cells were detectable in skin lesions from SSc women [105], other studies did not record a significant difference of male DNA in SSc women versus healthy controls [106,107].

Mammalian sex is determined by the inheritance of the sex chromosomes, XX in female and XY in male. The human Y chromosome contains a relatively low number of protein-coding genes (approximately 100), whilst the X chromosome encodes over 1000 proteins [108]. The X chromosome is therefore considered a greater source of potential mechanisms for sexual dimorphism. For example, men with Klinefelter’s syndrome (XXY) have 14-fold higher incidence of SLE than XY men [109]. Whilst altered hormone levels may be a contributing factor to these observations, a specific role for the X chromosome in autoimmunity was evidenced by the use of transgenic mice where the testes-determining *Sry* gene was deleted from the Y chromosome to yield XX and XY^-^ ovary-bearing mice. *Sry* was also reinserted on an autosome, yielding XX*Sry* and XY^-^*Sry* testes-bearing mice. Mice of each genotype were then gonadectomised, to remove the effects of sex hormones. In both models, the XX mice showed increased susceptibility to pristane-induced lupus, suggesting a role for X chromosome double dosage [110].

In order to maintain equal expression levels of X-encoded genes between males and females, mammalian females have evolved a dosage compensation mechanism known as X-chromosome inactivation (XCI). This involves the permanent inactivation of one of the X chromosomes early in embryogenesis. The choice of chromosome is random, and the transcriptional silencing of the inactivated X is clonally maintained in the developing cells, therefore females are mosaics of cells expressing either the maternal X or the paternal X [111]. Since the process occurs at random, theoretically females should then express X-linked mutations in only 50% of their cells, in contrast to 100% of a male’s cells, thereby protecting females from X-linked diseases. However, in some females XCI may be skewed, i.e., not a 50:50 balance of maternal and paternal X expressing cells, and this has been associated with several AD [111]. Additionally, XCI can be incomplete, leading to expression of some genes from both chromosomes. At least 15% of genes can escape XCI, and this may vary between cell types [112]. Some genes can also become reactivated on the inactive X chromosome and this has also been linked to the incidence of several AD [113,114,115].

The X chromosome is rich in immune-related genes including interleukin-1 receptor associated kinase 1 (IRAK-1), Toll-like receptor 7 (TLR7), Toll-like receptor 8 (TLR8), Forkhead box protein P3 (FOXP3), cluster of differentiation 40 ligand (CD40L), C-X-C Motif chemokine receptor 3 (CXCR3) and Bruton’s tyrosine kinase (BTK) [112]. Therefore, variations in expression of X chromosome regions due to XCI skew, escape or reactivation, could have important consequences for immunoregulation. The X chromosome is also rich in microRNAs (118, compared to just 2 on the Y chromosome), many of which have emerging and predicted roles in immunoregulation and fibrosis-linked signaling pathways [116]; mosaicism, skewing and incomplete XCI could also affect X-linked miRNA expression. Examples of known X-linked mechanistic contributions to AD are shown in Table 3.

Whilst sex hormones and the X chromosome are linked to CTD development via autoimmune mechanisms, their specific contribution to the pulmonary manifestations of these diseases have not yet been determined. Future studies examining pulmonary sex hormone signaling and X-linked genes/miRNAs in ILD incidence and progression may therefore identify new therapeutic targets.

## 7. Gender Differences in Clinical Research

It is clear that at a biochemical and cell level, sex hormones and gender differences play a role in disease development. However, there are under recognised gender biases to consider that can affect diagnosis, research and treatment, and therefore affect clinical outcomes. Much of the analysis of the IPF epidemiological data in the literature are retrospective and therefore susceptible to bias including referral bias. While it is recognised that women tend to seek healthcare earlier than men, in general as a group they are less likely to be referred to sub-specialty services [127]. In addition, even when referred to a respiratory service there is evidence for bias when using sex to make the diagnosis of an ILD. Assayag et al. conducted their own analysis of data from a large online evaluation of ILD clinical cases by an international group of respiratory physicians. In the original study, physicians were asked to give up to five diagnoses along with diagnostic likelihood in order to measure their level of confidence in their ILD diagnosis [128]. Results showed that there was a bias when sex was used to diagnose ILD, which could result in men being over diagnosed and women being underdiagnosed with IPF. Therefore, the exact prevalence of IPF in men and women may be difficult to estimate and subject to multiple sources of bias.

We conducted a review of the literature in the PubMed database (search carried out in April 2021) assessing gender distribution in randomised controlled trials and clinical trials over the last 10 years for CTD associated ILD and IPF separately (Supplementary data Figures S1–S4). Documents, meta-analysis, reviews, articles not published in English, trial protocols, research on the same trial participants in separate publications and missing gender data were excluded. This revealed how the male: female ratio of trial participants compared in each respiratory condition of interest. CTD has an estimated gender bias ratio of 7:1 to 10:1 female: male [65], but for participants in clinical trials with a diagnosis of CTD associated ILD the male: female ratio was much higher. Across 19 trials that reported gender demographic data, there were a total of 1982 female participants and 697 male participants enrolled, resulting in a ratio of 2.84:1 female: male. Similarly, IPF is estimated to have a gender bias ratio of 1:1.45, female: male [17], but across 134 clinical trials in the past ten years the female: male ratio of participants was 1:2.82. These findings may be confounded by pathophysiological factors (where not all those with CTD develop ILD); factors in the trial design, referral or recruitment bias; and behavioural differences of those who are willing to participate in trials (volunteer bias). Moreover, the epidemiological prevalence may be erroneous due to similar biases. Further to this, many trials failed to report demographics on gender, despite evidence of gender playing a significant role in disease development.

## 8. Discussion

Examination of sex differences in the current literature in ILD and CTD progression and development show that biological sex plays an important role in ILD, noted by the incidence and clinical outcomes of these diseases. Efforts to understand these differences have identified sex hormones to have diametric effects, e.g., estrogens enhance inflammation and androgens are immunosuppressive, whilst estrogens are protective against fibrosis and androgens appear more permissive of its progression [129]. This could impact the manifestations of ILD in males versus females, as females undergo considerable hormonal changes throughout their lifetime.

The X chromosome is another potential source of sex differences in disease pathology, e.g., some X chromosome genes may escape XCI in female cells, resulting at times in unfavourable immunoregulation. XCI in females can also be skewed in some immune cell populations, and a high level of skewing (e.g., more than 80% of cells expressing the same X chromosome) is associated with the incidence of numerous CTD. The mechanisms behind this effect are hypothesised to be a result of the loss of mosaicism in the dendritic cell (DC) population in the thymus, e.g., if the paternal X chromosome is preferentially inactivated in a high proportion of DCs, then the DCs expressing maternal X chromosome self-antigens will mainly tolerate the T cells. Autoreactive T cells specific for paternal X chromosome self-antigens therefore have a greater likelihood of escaping negative selection and entering the circulation, where they potentially lead to a breakdown in self-tolerance when they encounter their cognate self-antigens [111].

CTD have a higher female preponderance, but male sex seems to be a major risk factor for developing ILD in this cohort. Male CTD-ILD patients, especially in the presence of specific autoantibodies should be monitored closely for timely intervention with immunosuppressants and antifibrotic agents. Whilst our review focused on sex hormones and autoimmunity, development of ILD is also influenced by environmental factors as well as genetic background, which can trigger the expression of pro-inflammatory and pro-fibrotic factors, resulting in pulmonary fibrosis. Also of note, but not discussed in depth in this review, is the emerging evidence of sex-dependent differences in the ageing process, particularly the immune system. Whilst similar changes in T cell number and subset proportions occurs with age in both males and females, these changes occur significantly faster in males [130]. On the other hand, age-associated B cells (ABCs) appear to accumulate more in ageing females and are linked to AD pathogenesis [131]. XCI skew in females also increases with age, which may affect the onset of an AD or CTD [132].

Although we are beginning to decipher the causes and mechanisms behind pulmonary fibrotic diseases, we need to better understand the role of sex differences between the normal and diseased lung is required. In order for us to better understand sex differences not only in lung diseases but in all diseases, most of which have a bias towards a specific sex, researchers must define sex as an important experimental variable in their studies. A previous review examining the sex of cells and animals of all studies published in the AJP-Cell Physiology journal reported that approximately 20% of studies have used either cells or animals of male origin, 5% of female origin and in 75% of studies, sex was not reported [133]. Therefore, in the future, the sex of patient-derived cell lines, animals and/or human subjects used in these preclinical and clinical studies need to not only be recorded but we must ensure that we examine both sexes equally. In addition to leading to better research, this will offset the current bias seen, which in turn will aid in the discovery of new targets and potential therapies, paving the way for precision medicine.

Examining the gender balance of participants of IPF and CTD clinical trials over the last 10 years also demonstrates that the proportion of female participants recruited to each of the trials did not accurately reflect the estimated sex ratios of the disease in the general population. It is also important to point out that gender refers not only to the genetic, anatomical and physiological traits that a person is born with, but it is also influenced by societal, cultural and environmental factors. Gender is associated with differences in occupational activities and lifestyle that play significant roles in exposure to allergens, smoking, pollutants and pathogens along with differences in comorbidities, behavioural differences in how people seek medical care and bias in diagnosis and management. However, to date no clinical trials in ILD differentiate between biological sex and gender. A recent review by Sodhi et al. also touches on the importance of reporting the sex and gender of all subjects used in research, as well as the significance of including pregnant women in clinical trials [134]. With an increased emphasis from the majority of funding bodies for the purposeful consideration of gender and/or sex differences in the design of preclinical and clinical research, it is hoped that the contribution of each sex will become more balanced in future studies.

Future work will need to focus on deciphering the sex differences in ILD across its entire spectrum of presentations. Such work may help us to identify if responses to novel treatments are sex-dependent; evaluate if anti-androgens or estrogen replacement could serve as potential therapeutics; and aid in the elucidation of other novel targets, particularly targets that may have been overseen due to sexual bias in the subjects recruited for specific clinical trials. Such targets may include specific genes involved in the estrogen signaling pathway or those associated with the androgen receptor pathway. This in turn will hopefully lead to more effective interventions and better patient outcomes.

## Figures and Tables

**Table 1:** Epidemiological and clinical sex differences in IPF and selected CTD-ILD.

	Idiopathic Pulmonary Fibrosis	Rheumatoid Arthritis	Systemic Sclerosis	Systemic Lupus Erythematosus	Primary Sjögren’s Syndrome
**Gender ratio (F:M)**	1:1.5 to 2 [8,17]	2:1 [18]	3:1 [19]	9:1 [20]	9:1 [21]
**Affected age group**	>50 y	F: 50–60 y M: >70 y [22]	30–60 y [23] Males peak later	Late teens to early 40s [20]	Peaks in Females ~30 y and >55 y [24]
**Radiographic features**	UIP	UIP > NSIP [25]	NSIP > UIP [26]	NSIP, OP, UIP [27]	NSIP > UIP, OP, LIP [28]
**Lifetime Risk of ILD development**	ND	10% 9 times ↑ILD risk vs. people without RA [29]	Up to 90%, 40% have clinically significant ILD [30,31]	1–15% [32,33,34]	9–22% (F > M) [35,36,37]
**Potential risks for development of ILD**	Genetic predisposition, aging, male, smoking, GER, viruses [6,7]	RF [38], ACPA [39], MUC5B mutation [40], older age [41], male [41], smoking [42]	Anti-SCL70 and anti-Anti-U3RNP antibodies [43], Afro-Caribbean [44], male [45]	Anti-U1RNP [33], longstanding disease [46], older age [47], overlapping features with scleroderma [48]	Anti-SSA [49], older age, male, high CRP [50]
**Risk of mortality**	Survival worse in males [15], 3.75:1.5 per 100,000 M:F [51].	RA-ILD median survival 3-7y [41]. RA-UIP worse survival vs. RA-NSIP 10.18 vs. 13.62 y [52]	ILD associated mortality ↑ in males. Female increased mortality associated with PAH [45]	Higher in males [53]	4 fold after 10 y of disease compared with those without lung involvement [54]

Usual interstitial pneumonia (UIP), Not determined (ND), gastroesophageal reflux (GER), non-specific interstitial pneumonia (NSIP), Interstitial lung disease (ILD), rheumatoid factor (RF), anti-citrullinated protein antibodies (ACPA), RA-associated interstitial lung disease (RA-ILD), Pulmonary arterial hypertension (PAH), Organising Pneumonia (OP), Lymphocytic Interstitial Pneumonia (LIP), increase (↑).

**Table 2:** Sex hormone effects on CTD pathophysiology.

Hormone	Alteration or Mechanism	Ref.
**SLE**		
Estrogen	Allows autoreactive B cells to escape tolerance mechanisms	[90]
	Promotes IFN-γ production	[91]
	Upregulates expression of intracellular (but not surface) Toll-like receptors	[92]
	Increased plasma estradiol in women with SLE versus healthy women; estradiol reduces FOXP3 expression in regulatory T cells	[93]
Androgens	Testosterone suppresses anti-dsDNA antibody production	[94]
	Women with SLE have lower plasma androgen levels than healthy women	[95]
	Men with SLE have low testosterone (and elevated 16-hydroxyestrone, estrone and luteinizing hormone)	[96]
**Sjögren’s Syndrome**		
Androgens	Protective against disease development—gonadectomy of non-obese diabetic (NOD) mice exacerbated the disease	[97]
**Rheumatoid Arthritis**		
Estrogens	Males with RA have increased circulating levels of estradiol and decreased estrone	[98]
	Males and females have increased synovial fluid estrogens relative to androgens which may contribute to synovial cell proliferation, e.g., macrophages and fibroblasts	[99]
Androgens	Males with RA have decreased testosterone and dehydroepiandrosterone	[98]
**Systemic Sclerosis**		
Estrogens	Increased estradiol in older men with dcSSc, associated with decreased survival	[100]
	Estradiol may augment fibroblast dysfunction	[101]

**Table 3:** X chromosome links to the pathogenesis of CTD.

Disease	Mechanism	Refs.
**SLE**	X inactivation specific transcript (XIST), the non-coding RNA which orchestrates XCI, was significantly upregulated in SLE patients, together with skewed XCI in lymphocytes	[117]
	T cells from women with SLE showed overexpression of 18 X-linked miRNAs; males with SLE did not show overexpression of any	[118]
	BTK (an X-linked gene) promotes activation, plasma cell differentiation, and class switching of autoreactive B cells	[119]
	CD40L reactivation on the inactive X chromosome in T cells of females with SLE	[113]
**RA**	Higher incidence of skewed XCI in RA patients	[120,121,122]
	TLR7 can escape XCI, resulting in increased gene dosage	[123]
	CD40L reactivation on the inactive X chromosome in T cells of females with RA	[114]
**SSc**	Higher incidence of skewed XCI in SSc women v healthy women; association with reduced FOXP3 expression in Tregs	[120,124,125,126]
	CD40L reactivation on the inactive X chromosome in T cells of females with SSc	[115]

## Supplementary Materials

The following supporting information can be downloaded at: https://www.mdpi.com/article/10.3390/biomedicines10123030/s1, Figure S1: Publication of clinical trials and randomised control trials (RCTs) in PubMed across the past 10 years; Figure S2: Publication of clinical trials and RCTs in PubMed with the term “idiopathic pulmonary fibrosis” in the title/abstract across the past 10 years; Figure S3: Summary of how the literature review was conducted in order to assess gender distribution for CTD associated ILD; Figure S4: Summary of how the literature review was conducted in order to assess gender distribution for ILD in the PubMed database.
