# Supplementary material for: Sexual Dimorphism in Interstitial Lung Disease
Source: Biomedicines. 2022 Nov 24;10(12):3030. doi: 10.3390/biomedicines10123030 (PMC9775147; doi:10.3390/biomedicines10123030)
Supplement: Supplementary file 1 [file biomedicines-10-03030-s001.zip › biomedicines-2014906-supplementary.pdf]

Supplementary Materials

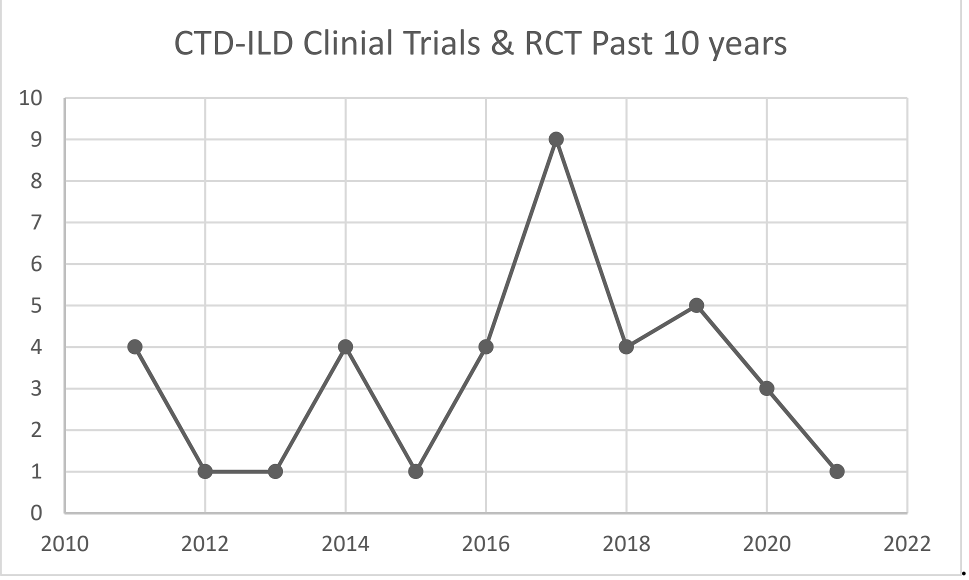

Figure S1. Publication of clinical trials and randomised control trials (RCTs) in PubMed across the past 10 years.

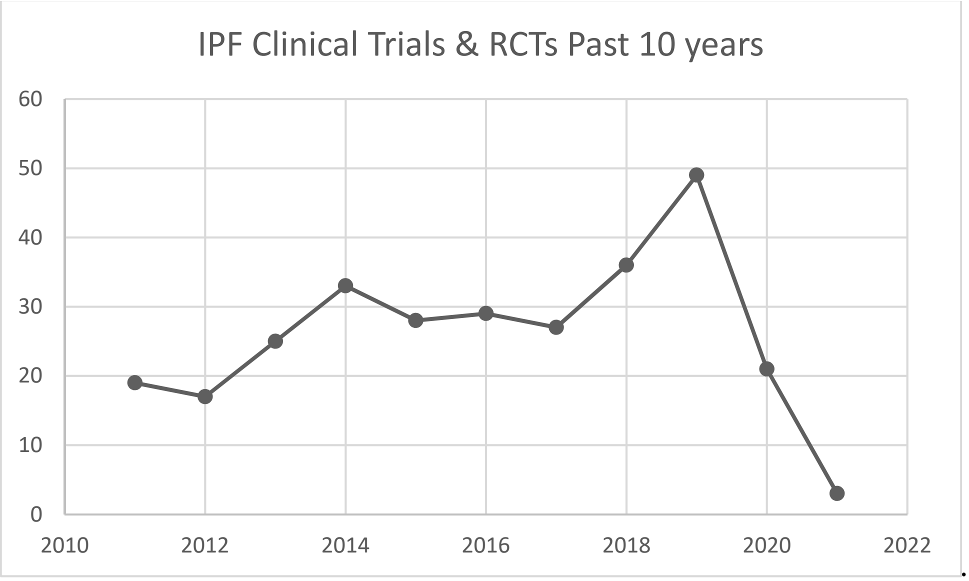

Figure S2. Publication of clinical trials and RCTs in PubMed with the term "idiopathic pulmonary fibrosis" in the title/abstract across the past 10 years.

CTD-ILD PRISMA

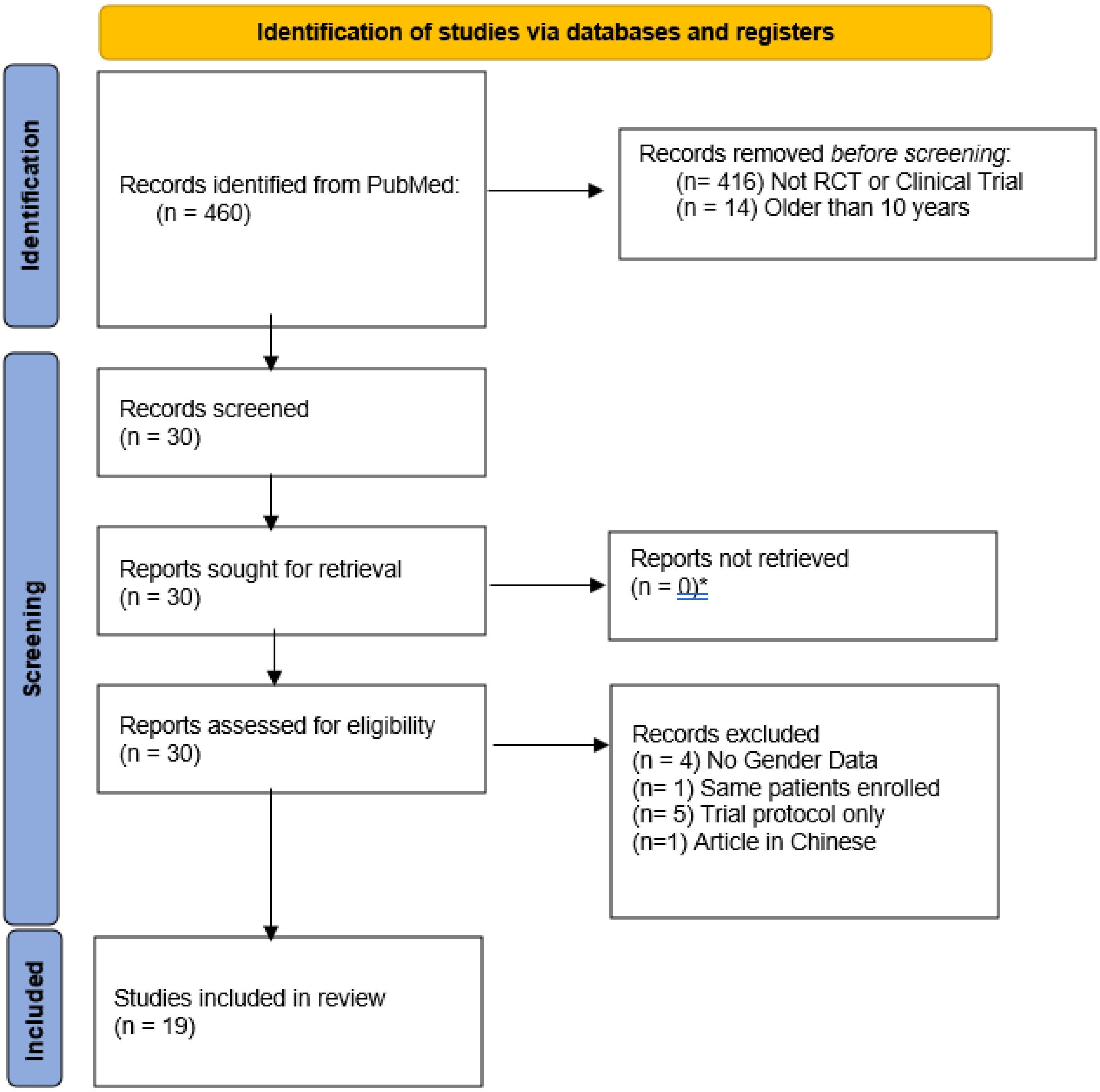

Figure S3. Summary of how the literature review was conducted in order to assess gender distribution in randomised controlled trials and clinical trials over the last 10 years for CTD associated ILD in the PubMed database.

CTD-IPF PRISMA

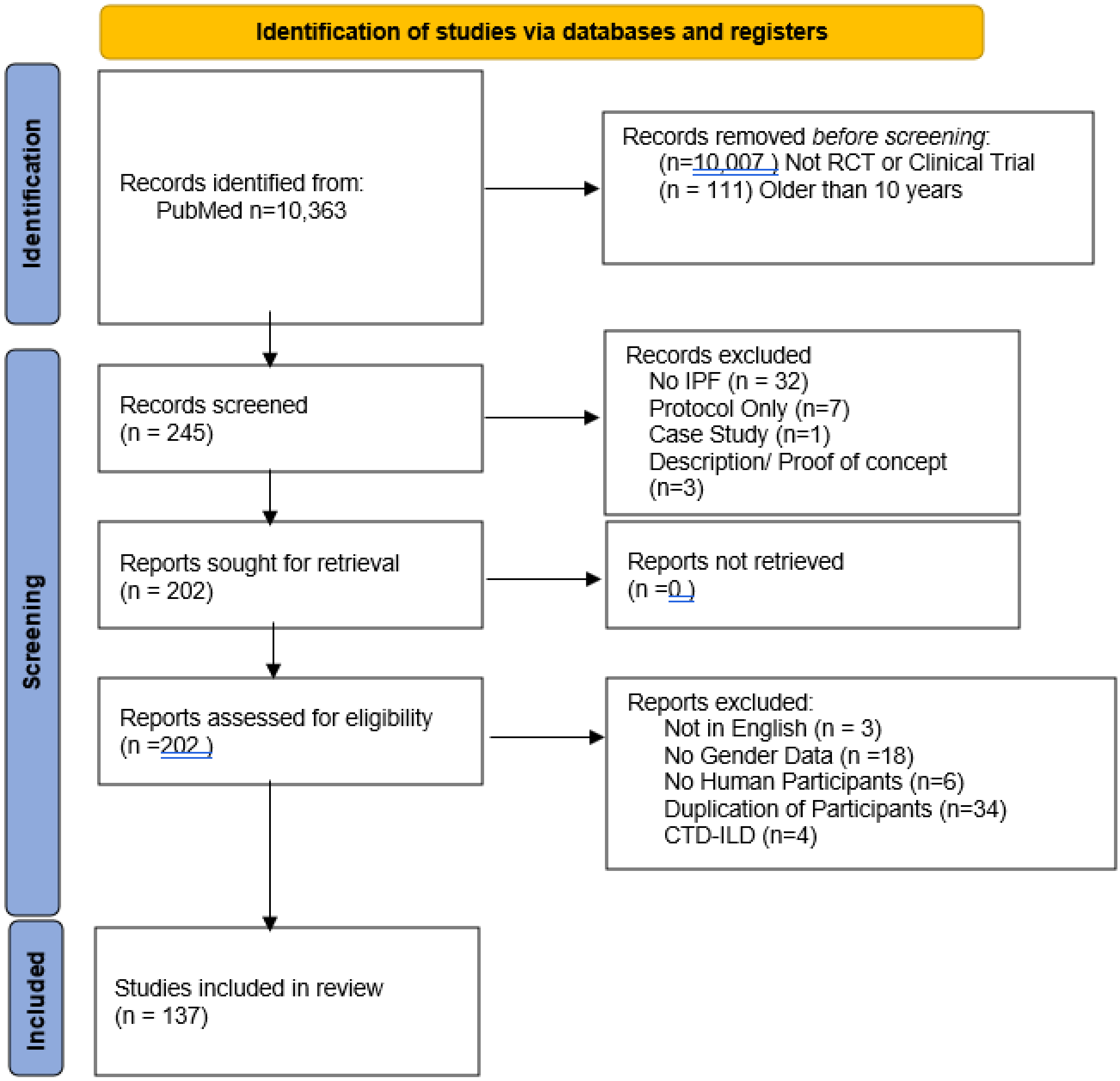

Figure S4. Summary of how the literature review was conducted in order to assess gender distribution in randomised controlled trials and clinical trials over the last 10 years for ILD in the PubMed database.
